# Virtual Reality Images of the Home Are Useful for Patients With Hospital-Based Palliative Care: Prospective Observational Study With Analysis by Text Mining

**DOI:** 10.1089/pmr.2023.0017

**Published:** 2023-08-07

**Authors:** Tomoyo Mukai, Yoshi Tsukiyama, Shinobu Yamada, Akinori Nishikawa, Shinya Hayami, Rie Noguchi, Junko Yoshida, Maki Kashiwada, Shigeru Ohta, Toshio Shimokawa, Hiroki Yamaue

**Affiliations:** ^1^ Departments of Nursing, Wakayama Medical University Hospital, Wakayama, Japan.; ^2^ Palliative Care Center, Oncology Center, Wakayama Medical University Hospital, Wakayama, Japan.; ^3^ The Department of Anesthesiology, Wakayama Medical University, Japan.; ^4^ Wakayama Medical University Graduate School of Health and Nursing Science, Wakayama, Japan.; ^5^ Division of Blood Transfusion, Wakayama Medical University Hospital, Wakayama, Japan.; ^6^ Departments of Hematology/Oncology, Wakayama Medical University Hospital, Wakayama, Japan; ^7^ Division of Medical Informatics, Wakayama Medical University Hospital, Wakayama, Japan.; ^8^ Second Department of Surgery, Wakayama Medical University, Wakayama, Japan.; ^9^ Wakayama Medical University, School of Pharmaceutical Sciences, Wakayama, Japan.; ^10^ Clinical Study Support Center, Wakayama Medical University Hospital, Wakayama, Japan.; ^11^ Department of Cancer Immunotherapy, Wakayama Medical University, Wakayama, Japan.

**Keywords:** cancer, palliative care, virtual reality

## Abstract

**Background:**

Malignancy patients who need long-term hospitalization can feel loneliness affecting their quality of life. The global COVID-19 pandemic has caused visiting restrictions that could mean patients who might be missing out on family support and palliative care, therefore, need to adapt and change. We used virtual reality (VR) technology with the aim of reducing feelings of loneliness among these patients.

**Objectives:**

In a small cohort setting, we aimed to clarify the usefulness of VR viewing for this purpose by text mining interviews with the patients in palliative care after their VR experience, and to clarify the feasibility of this program.

**Design and Setting/Subjects:**

Four consecutive Japanese patients in the palliative care unit viewed personalized familiar persons or places through VR goggles, while communicating by telephone. After the VR experience, text mining of the patients' interviews was used to extract the words for the frequency count and co-occurrence analysis.

**Results:**

Four clusters were extracted: “relief from the pain of hospitalization by feeling safe and secure with family members nearby,” “using VR to regain daily life,” “immersive feeling of being in the same space as family,” and “loneliness due to the realistic feeling of separation from the family through VR experience.” There were no cases of VR sickness.

**Conclusion:**

Our results attained by text mining suggest the promising potential of VR imaging of familiar surroundings for patients in palliative care.

## Background

The global spread of COVID-19 has resulted in limitations of family visitations of hospitalized patients and created an unprecedented situation of widespread patient social isolation. In this context, malignancy patients requiring long-term hospitalization are thought to have felt loneliness that severely impacted upon their quality of life. Such patients are likely to be missing out on support and encouragement from their family and friends. Palliative care has, therefore, been significantly altered by the COVID-19 pandemic.^1,2^

## Objectives

Virtual reality (VR; a computer-generated environment with scenes and objects that appear to be real) is proposed in this study as a means of alleviating the social restriction and isolation caused by the pandemic. The application of VR technology has been previously applied to palliative care.^3–5^ Experience of an extraordinary or fantasy world by VR software reportedly improved patients' pain and symptoms according to Edmonton symptom assessment system score.^6,7^ VR technology may also have the potential to relieve symptoms such as pain and anxiety for the patients in palliative care.^8^

To facilitate the application of information and communication technology for malignancy patients necessary for palliative care, we assembled a multidisciplinary project team (doctors and nurses in the palliative care unit, system engineers, and statisticians) in our hospital. We hypothesized that the application of VR technology in this setting will aid patients' feelings—not by creating a virtual experience of a new unusual environment for the patients, but rather by creating a virtual experience of a familiar daily environment personal to the patient. We aimed to clarify the usefulness and feasibility of this program in a small cohort through text mining of interviews with the patients after a short experience of this VR application.

## Design and Setting/Subjects

### Patients

Enrolled in this study were four consecutive Japanese malignancy patients whose terminal state required enrollment in the palliative care unit of our hospital between November 2021 and June 2022. This period was during the COVID-19 worldwide pandemic, when it was still considered by the World Health Organization to be a public health emergency of international concern. Selection criteria were as follows: (1) desire to go home but that not being possible due to the patients' condition, (2) sufficient visual acuity to recognize the VR images, and (3) having family or friends who could take pictures in and around the patient's home environment.

The exclusion criterion was patients who did not consent to this study and those who had difficulty in answering the interview survey. The study protocol was approved by Wakayama Medical University Ethical Committee (approval number: 3165).

### VR images of friendly place/subject for the patients

Family members of the patients were asked to take videos of themselves at home and other familiar places where the patients wished to visit using a 3D camera (RICOH THETA V, RICHO, Tokyo, Japan). Recorded videos could then be shown to the patients through VR goggles (OculusQuest2, Meta, Menlo Park, CA). While viewing these recorded videos, the patients communicate with their family members or friends by telephone. Program participation could be repeated if the patients desired.

### Survey method

Surveyed variables included patients' characteristics such as age, gender, disease (type of malignancies), and Eastern Cooperative Oncology Group (ECOG) performance status (PS).^9^ We also recorded information on video recording circumstances, such as shooting location, participating family members, and viewing time. To observe and evaluate emotional changes after the initial video sharing, the patients were interviewed about their hospitalization and the VR intervention subsequently 15 minutes after VR viewing, loosely following a 10-item interview guide (Table 1), over ∼30 minutes in a private room where the patients' privacy could be secured. This 10-item interview guide was newly constructed by nurses in the palliative care unit especially for the following text mining method.

**Table 1.** Ten-Item Interview Guide for the Distress of Hospitalization and Virtual Reality Intervention

1. How did the VR experience make you feel? 2. Specifically, how did the VR experience make you feel about the distance between you and your family/friends? 3. How do you feel about your family/friends through the experience of VR? 4. How was your communication with your family/friends during the VR experience? 5. How about your feeling at home through the VR experience? 6. When do you feel the distress in your hospitalization? 7. How about the distress during VR? 8. How do you feel reduce of the distress? 9. How about the distress after experience of VR? 10. How about your hope of enjoying VR again?

VR, virtual reality.

### Text mining to evaluate the usefulness of VR imaging using word frequency analysis and co-occurrence network

Text mining is a method of extracting useful information from a large amount of text data using natural language processing techniques.^10^ An approach of text mining for palliative care was gradually spread.^11–13^ Text Mining Studio Ver. 6.4 (NTT Data Mathematical Systems, Inc., Tokyo, Japan) was used in this study. A verbatim transcript was created from the interview content to grasp the purpose and context, and the narratives then considered to be related to the distress of hospitalization were highlighted as text data. Words with similar meanings were analyzed after appropriate organization.

As the definition of the frequency count, words used more than twice were determined within the entire text. Words with co-occurrence relationships were automatically grouped together from the entire text and classified into clusters. The frequency of extracted words can be visually confirmed as the size of a circle, and their relevance (co-occurrence) as a line connection.^10^ In addition, cancer-certified nurse specialists were asked for their opinions to ensure the reliability and validity of the data.

## Results

Patient characteristics and video recording status are given in Table 2. Four consecutive malignancy patients were enrolled into this study, two males and two females. Their conditions were relatively poor because of the progression of malignancies. Three patients were ECOG PS 4, suggesting necessity of hospitalization. Shooting location familiar to the patients was homes and workplaces, while participating family members were spouses, parents, children, siblings, grandchildren, and pets or friends. The patients watched the recorded video for 5–15 minutes by VR goggles and complained of no VR sickness such as vertigo, nausea, or discomfort.

**Table 2.** Patients' Characteristics and Video Recording Status

	Age	Gender	Disease (type of malignancies)	PS	Shooting location	Participating family members	Viewing time (minutes)
A	30's	Male	Synovial sarcoma	4	Parent's home	Parents, sister, dog	15
B	60's	Male	Myelodysplastic syndrome	0	Home	Wife, children, grandchildren	15
C	50's	Female	Middle thoracic esophageal cancer	4	Home	Husband, children, cat,	15
D	50's	Female	Breast cancer	4	Home, workplace	children	5

PS, performance status.

**FIG. 1.**
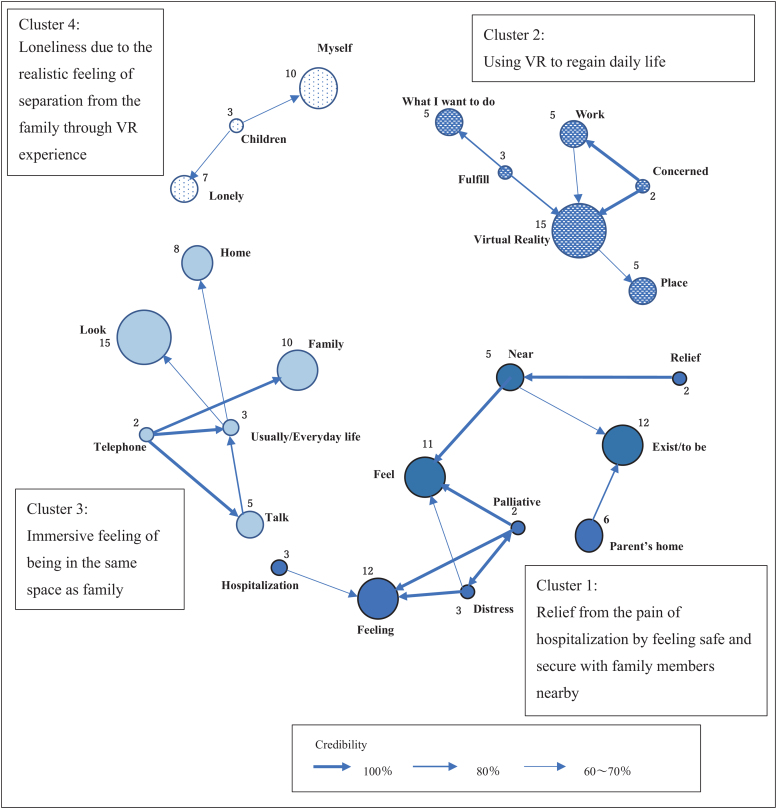
Co-occurrence network. Closed circle corresponded to the frequency of words from the interview. The numbers around the circle represented the frequency of each word. Arrow indicated the direction between two words and its thickness expressed the confidence/credibility. VR, virtual reality.

Shown in Table 3, the most frequently appearing words (English words here representing Japanese counterparts) from the interview were “VR,” “look,” “feeling,” “exist/to be,” “feel,” “family,” “myself,” “pet,” “home,” and “well.” As co-occurrence network analysis, four clusters were extracted (Fig. 1). For the first cluster (relief from the pain of hospitalization by feeling safe and secure with family members nearby), the following words were extracted: “feel,” “exist/to be,” “near,” “distress,” “hospitalization,” and “relief.” Strong co-occurrence was shown among “relief,” “near,” “feel,” “distress,” “palliative,” and “feeling.”

**Table 3.** Number of Occurrences of Selected Words Related to Alleviating the Pain of Hospitalization

Extractive words			Extractive words			Extractive words		
Reading words (Japanese)	Meaning of words (English)	Count	Reading words (Japanese)	Meaning of words (English)	Count	Reading words (Japanese)	Meaning of words (English)	Count
VR	Virtual Reality	15	shigoto	Work	5	tanoshii	Enjoyable	2
miru	Look	15	basho	Place	5	tanoshimu	Enjoy	2
kimochi	Feeling	12	haiguusha	Spouse	5	yurumu	Palliative	2
iru	Exist/to be	12	hanasu	Talk	5	kaeru	Go home	2
kanjiru	Feel	11	satsuei	Filming	4	kininaru	Concerned	2
kazoku	Family	10	kanau	Fulfill	3	iu	Say/tell	2
jibun	Myself	10	ureshii	Happy	3	kangaeru	Think	2
pet	Pet	8	kodomo	Child	3	iku	Go	2
jitaku	Home	8	tsurasa	Distress	3	sanpo	Have a walk	2
yoi	Well	8	nyuuin	Hospitalization	3	nagai	Long	2
sabishii	Lonely	7	byouki	Sickness	3	denwa	Telephone	2
jikka	Parent's home	6	fudan	Usually/everyday life	3	uru	Deal in	2
kibou	What I want to do	5	warui	Negative/bad	2	nakunaru	Pass away	2
chikaku	Near	5	anshin	Relief	2			

Then, the following text was extracted: “I felt like that I was in the center of my parents' home,” “I felt close and the pain of feeling was relieved,” and “I felt safe because I felt close to my family.”

In the second cluster (using VR to regain daily life), featured words were “VR,” “place,” “work,” “what I want to do,” “fulfill,” and “concerned.” Strong co-occurrence relationships were shown between “VR,” “concerned,” and “work” and “VR,” “what I want to do,” and “fulfilled.” The following text was, therefore, extracted: “I was concerned about whether my job would be alright,” “Everything was not as I hoped, but there was a possibility that some of my wishes could be fulfilled by VR,” and “There were surely places where my family could visit or not, but I thought that I could fulfill what I really wanted my family to do for me.”

In the third cluster (immersive feeling of being in the same space as family), featured words were “look,” “family,” “usually/everyday life,” “talk,” “telephone,” and “home.” Strong co-occurrence relationships were shown in “talk,” “usually/everyday life,” and “family.” As the extracted sentence, “I talked on the phone with my family with feeling closer and talked more than I usually do on the phone.”

In the fourth cluster (loneliness due to the realistic feeling of separation from the family through VR experience), featured words were “myself,” “lonely,” and “children.” Then, the following text was extracted: “My spouse was kind, and I also missed him/her, and I had to say good-bye and similar to my children” and “I was worried that I would no longer be in this world and that I might be forgotten.”

## Conclusions

We demonstrated the promising potential of VR imaging by text mining interviews with patients in palliative care taken after a VR experience of their familiar home surroundings. Moreover, the feasibility of this program might be indicated by no VR sickness in any of the four patients.

As indicated in Figure 1 by Cluster 1 (relief from the pain of hospitalization by feeling safe and secure with family members nearby), especially during the COVID-19 pandemic, patients who had to be separated from their families and society expressed “the feeling of being in the center of my parents' home,” “home,” and “relief at feeling close to my family.” Simultaneous sharing of VR images of their family members and friends at home or familiar places made the patients feel immersed as if there and the psychological change was “less painful.” These results supported the findings of the previous studies.^3,4^

According to Cluster 2 (using VR to regain daily life), the patients expressed the promising possibility of VR and their desire for their concerns about daily life lost due to hospitalization to be heard. These extracted sentences suggested a high feeling of desire for the place of memories and daily life among the patients. Recovering aspects of a patients' daily life through VR goggles might create heightened awareness of their own existence in connection with familiar people and allow them to maintain their own identity.^14^ Next, as shown by Cluster 3 (immersive feeling of being in the same space as family), the patients felt greater immersion by viewing VR and speaking in real time on the telephone as if they were with family in the same place.

Real-time sharing of both space and time could facilitate better communication than a conversation using only tablets. Cluster 4 (loneliness due to the realistic feeling of separation from family through VR experience) indicated that the patients were thought to feel distress from confronting the reality in which the patients themselves exist at home.^15^ When these patients with terminal cancer noticed the disconnection of the relationships with others, separation from the outside world, or isolation from familiar people and things, they reported experiencing an unrelieved loneliness and anxiety.^16^

In this study, viewing VR images of familiar people and surroundings provided an opportunity for the patients to express their spiritual pain. Generally in malignancy patients with terminal state, the greatest support for spiritual pain is thought to be empathy and listening.^17^ VR viewing might contribute to understanding of thoughts and feelings that the patients had not expressed and thus allow understanding of the patient's distress that may improve their care. Viewing VR images of the familiar people and surroundings as a cause of spiritual pain has not been previously reported.

The emotional bond to familiar places can bring the feeling of comfort and security, while the loss of these important bonds was said to destabilize the patient's self-concept.^18^ It is thus essential for medical personnel to understand the emotional changes for the patients, good and bad, through the VR experience, as well as to give consideration to allow the patients to safely express various feelings and emotions.

This study was, however, a small cohort, and there were several limitations. First, only four patients were enrolled in this study, so further trials including larger number of patients are needed. Second, the final goal of this study would be to realize palliative care in which hospitalized patients themselves feel connected to society by provision of VR images of the home, workplaces, and other environments that were part of the daily life before their hospitalization, and to allow real-time conversations with their family and friends using sophisticated VR technology. Third, this VR technology is currently unidirectional delivery from the family to the patients, bidirectional dialogue between the patients and family might have different results.

Viewing VR imaging of the familiar people and surroundings might have promising potential for the patients undergoing palliative care. Text mining of patient interviews after their VR experience elucidated some specific patient thoughts and concerns.

## Abbreviations Used

ECOG PS: Eastern Cooperative Oncology Group performance status
VR: virtual reality
